# Wound Closure Outcomes Suggest Clinical Equivalency Between Lyopreserved and Cryopreserved Placental Membranes Containing Viable Cells

**DOI:** 10.1089/wound.2019.1028

**Published:** 2019-10-16

**Authors:** Charles E. Ananian, R. Daniel Davis, Eric L. Johnson, Matthew J. Regulski, Alexander M. Reyzelman, Molly C. Saunders, Alla Danilkovitch

**Affiliations:** ^1^ New Hope Podiatry, Los Angeles, California.; ^2^ Family Podiatry Center, Bridgeport, Connecticut.; ^3^ Bozeman Health Deaconess Hospital, Wound and Hyperbaric Center, Bozeman, Montana.; ^4^ Ocean County Foot & Ankle Surgical Associates, P.C., Forked River, New Jersey.; ^5^ California School of Podiatric Medicine at Samuel Merritt University, Oakland, California.; ^6^ Osiris Therapeutics, Inc., Columbia, Maryland.

**Keywords:** viable, lyopreserved, placental membrane, nonhealing, wound

## Abstract

**Objective:** To evaluate the clinical outcomes of lyopreserved placental membrane containing viable cells (vLPM) in the treatment of nonhealing wounds of various etiologies, and to compare them to those previously reported for cryopreserved placental membrane containing viable cells (vCPM).

**Approach:** Patients with nonhealing wounds who qualified to receive advanced wound therapies were consecutively enrolled and treated weekly with vLPM plus standard of care (SOC) at five centers. Data were de-identified and retrospectively analyzed. Outcomes included closure, time to closure, number of vLPM applications, and adverse events (AEs).

**Results:** Seventy-eight patients with 98 wounds (41 diabetic foot ulcers [DFUs], 19 venous leg ulcers [VLUs], 10 surgical, and 28 others) with an average size of 13.3 cm^2^ and 8.7 months duration were treated. Fifty-eight of the 98 wounds (59.2%) achieved complete closure with median time to closure of 63 days and 6 vLPM applications. The closure by wound etiology was 63% for DFUs, 47% for VLUs, 70% for surgical wounds, and 57% for other types of wounds. Similar closure rates have been previously demonstrated for vCPM. Wound duration was the main predictor of closure: 65.8% versus 30.0% (*p* = 0.004) closure was achieved for wounds of ≤12 and >12 months duration, respectively. There were no AEs related to vLPM application.

**Innovation:** This is the first multicenter case series evaluating the clinical outcomes of vLPM in a real-world setting.

**Conclusion:** These results support clinical equivalency between the two placental membrane formulations with the added convenience of room-temperature storage for vLPM, allowing it to be used in any wound-care setting.

**Figure:**
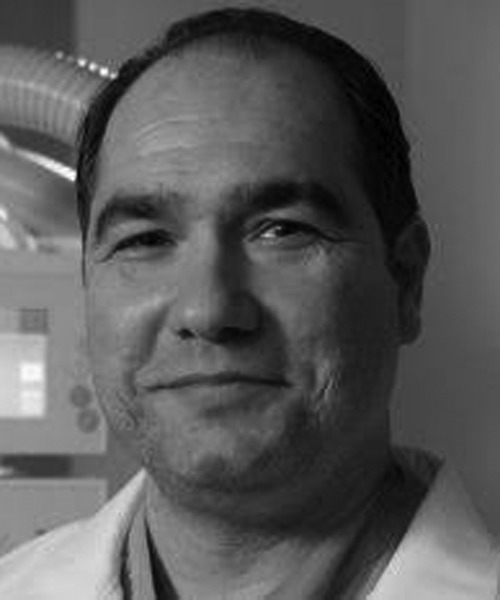
**Charles E. Ananian, DPM**

## Introduction

Chronic or nonhealing wounds are defined as wounds that are unable to proceed through the normal phases of healing in a timely and orderly manner.^1^ Approximately 2% of the United States population suffers from a nonhealing wound, presenting a significant therapeutic challenge to wound-care providers and a high cost burden to our health care system and society.^1,2^ Often, nonhealing wounds are linked to underlying patient comorbidities such as diabetes mellitus (DM), obesity, venous insufficiency, and arterial disease, all of which negatively impact wound healing.^3^

Current standard of care (SOC) for nonhealing wounds typically includes cleansing and debridement of necrotic and infected tissue, establishment of adequate circulation, maintenance of a moist wound environment, nutritional support, infection management, and offloading or compression depending on wound location and etiology.^4^ However, in patients with significant comorbidities, SOC alone often is not sufficient for wound management. As a result, advanced adjunctive therapies are recommended for these difficult-to-heal wounds. Skin substitutes, one class of advanced wound-care therapies, include different categories, such as bioengineered matrices, xenografts, and tissue allografts. Human placental membranes have a long history in wound management.^5,6^ Recently, with advances in preservation technologies, many different placental tissue allografts have become commercially available, including a cryopreserved placental membrane containing viable cells (vCPM).^6,7^

In vCPM, which can be derived from amnion or chorion, all of the components of fresh placental tissue are preserved including the three-dimensional collagen-rich matrix, endogenous growth factors, and viable cells.^7^ Preservation of all components allows retention of the anti-inflammatory, antifibrotic, antimicrobial, and angiogenic properties inherent to fresh placental tissue.^8–11^ High closure rates for difficult-to-treat wounds of different etiologies and locations have been demonstrated in multiple vCPM clinical studies.^12–20^

Two prospective studies, one utilizing amnion-derived vCPM and one utilizing chorion-derived vCPM, demonstrated a 62% and 59.3% closure rate for chronic diabetic foot ulcers (DFUs) and chronic complex DFUs, respectively.^12,14^ Results of these DFU prospective clinical trials are mirrored by reported DFU closure rates in real-world patients. In 2018, Raspovic *et al.* analyzed data from a wound care registry and reported a 59.4% wound closure rate with amnion- and chorion-derived vCPM.^16^ Multiple vCPM case series' in different patient populations, such as peripheral arterial disease (PAD) smokers, patients with surgical tract wounds, or with exposed hardware, have reported high and durable closure rates.^18–20^ In two retrospective studies, various types of wounds managed with vCPM plus SOC showed closure rates of 76.1% and 63.0%, respectively.^13,15^ In a prospective, single-arm study, 53.0% of patients achieved complete wound closure with vCPM plus SOC in the treatment of chronic venous leg ulcers (VLUs) that had previously failed 12 weeks of SOC.^17^ All these studies show similar closure rates for wounds of various etiologies and locations that have been managed with either amnion- or chorion-derived vCPM adjunct to SOC.

Until recently, cryopreservation was the only technique able to preserve viable cells and tissue for long-term storage. While cryopreservation allows for the long-term storage of tissue containing viable cells, the need for specialized equipment to maintain ultralow temperatures for shipment and storage limits the use of cryopreserved product to medical facilities that have deep freezers.^21^ Advances in preservation techniques, however, have led to the development of a lyopreservation method that allows for viable tissues to be stored at room temperature. Using this lyopreservation technique, a new formulation of placental membrane, has recently been developed.

Similar to vCPM, lyopreserved placental membrane containing viable cells (vLPM) retains all the components of the native tissue.^22^ A recent scientific study demonstrated that the vLPM extracellular matrix structure, levels of growth factors, and percent of endogenous viable cells were similar to those of vCPM and fresh amniotic membrane.^23^ vCPM and vLPM are tissue allografts categorized as cellular and/or tissue-based products and are intended for use in the management of acute and chronic wounds. The key difference between the two formulations is the required storage and shipment temperature: vCPM is stored at −75°C to −85°C and currently has a 3-year shelf-life at this temperature, whereas vLPM can be stored at room temperature with a current shelf-life of 1 year.^7,22^ With new data, the shelf-life for both products is expected to be extended in the future.

The purpose of this study was to evaluate clinical outcomes of vLPM in patients with nonhealing wounds of various etiologies and locations and compare them to outcomes previously reported for vCPM.

## Clinical Problem Addressed

Often, difficult-to-heal wounds require advanced therapies when first-line SOC treatment has failed. vCPM has been on the market since 2010 and has demonstrated positive clinical outcomes in the treatment of various types of wounds. However, for shipment and storage, vCPM requires specialized ultralow temperature equipment that limits the use of vCPM to medical facilities that have such equipment. A new formulation of placental membrane preservation using a new lyophilization technique that allows viable tissues to be stored at room temperature has been developed. Similar to vCPM, the vLPM retains all components and properties of native tissue. vLPM, however, is conveniently stored at room temperature. This study shows positive clinical outcomes with vLPM use for nonhealing wounds of different etiologies and locations, and shows wound closure rates similar to those previously reported for vCPM. The provided evidence addresses an important question for wound-care providers and payers regarding vLPM clinical performance and its comparability to vCPM.

## Materials and Methods

### Study design and patient population

This was an open-label case series evaluating the clinical and safety outcomes of vLPM in the treatment of nonhealing wounds of various etiologies at five different centers across the United States. Seventy-eight patients with 98 wounds who were qualified to receive advanced wound therapies between December 2017 and February 2019 were consecutively enrolled and treated with vLPM plus SOC. To be eligible, patients were 18 years or older with a nonhealing wound of any etiology and location, and had previously failed SOC treatment. Nonhealing wounds were defined as wounds with no progression toward closure with 4 weeks of SOC, or wounds in patients with significant comorbidities that put them at a high risk for nonclosure. Exclusion criteria included participation in any other skin substitute studies, active index wound infection, and alcohol and drug abuse.

Written informed consent was obtained from each patient before data analysis. Patient medical charts were the source of data. Data from the five centers were de-identified, consistent with the terms and conditions outlined in the Health Insurance Portability and Accountability Act of 1996 (HIPAA), and were pulled and analyzed retrospectively. This study was conducted in compliance with the ethical rules outlined in the Declaration of Helsinki. Due to retrospective analysis, this study was Institutional Review Board (IRB) exempt.

### vLPM description and treatment regimen

vLPM (GrafixPL PRIME^®^; Osiris Therapeutics, Inc., Columbia, MD) is aseptically processed from donated human placental tissue following rigorous quality assurance standards and is stored and distributed for use in accordance with the regulations outlined in 21 Code of Federal Regulations (CFR) 1271 and the standard of the American Association of Tissue Banks (AATB). All donors have been extensively screened and all tissues have been recovered, processed, stored, tested, and distributed in accordance with current U.S. Federal Regulations, current AATB standards, and state/local regulations as required. Every lot is tested per United States Pharmacopeia (USP) <71> sterility tests, residual moisture content analysis per USP <921> water determination, and custom *in vitro* assays to determine the presence of epidermal growth factor and the presence of viable cells (mesenchymal stem cells, epithelial cells, and fibroblasts) across ≥70% of the tissue tested.^22^ Comprehensive analyses of structural and functional properties of vLPM have been performed and reported previously.^23–25^

Weekly applications of vLPM were recommended and three different sizes of vLPM were used during the course of treatment: 5 × 5 cm, 3 × 4 cm, and 16 mm. Before product application, wounds were cleaned and debrided per investigator discretion. Following vLPM application, a nonadherent dressing was applied. Patients with DFUs were required to wear an offloading device, such as a standardized fixed ankle walker or postoperative shoe. Patients with VLUs were required to receive multilayer compression. Patients were instructed to leave dressings dry and intact. Treatment progress and safety evaluations were performed at each visit until the patient achieved complete closure or until the investigator terminated vLPM use. Up to 12 weekly applications of vLPM were recommended, however, at the discretion of the investigator, more applications could be used if a wound was progressing toward closure.

### Clinical outcomes and statistical analyses

The primary endpoint was the proportion of patients who achieved complete wound closure (defined as 100% reepithelialization as determined by the investigator) by the end of treatment. Other endpoints included time to closure, number of vLPM applications, percent area reduction (PAR) for nonclosed wounds, and treatment-related adverse events (AEs).

Subanalyses of closure outcomes for wounds ≤ and >12 months duration were performed, as well as outcomes for wounds ≤ and >3.62 cm^2^ (small and large). The median wound size, 3.62 cm^2^, was selected as a cutoff between small and large wounds.

Descriptive statistics for continuous variables included the mean, standard deviation, median, and ranges. Statistics for categorical variables included frequencies and percentages. Evaluation of bivariate associations between the primary endpoint (wound closure) and other study variables were also performed. A *χ*^2^ test was used for binary and categorical variables, and a one-way analysis of variance (ANOVA) test was used for associations between continuous study variables and the primary endpoint (wound closure). A linear regression model was used to assess an association between the time to closure outcome and continuous variables (age, wound size, and wound duration). A *p*-value of ≤0.05 was considered significant.

## Results

### Patient demographics and wound characteristics

Of the 78 patients treated with vLPM, 56 (71.8%) were male and 22 were female with an average age of 62.7 years (range 24–94). Patient comorbidities included 63% DM, 42% venous insufficiency, 47% hypertension, 14% chronic kidney disease, 13% PAD, 12% hyperlipidemia, 9% lymphedema, and 6% end-stage renal disease. Fifty-one of the 78 patients (65.4%) had two or more comorbidities. Furthermore, 11 of the 78 patients (14.1%) were receiving treatment for multiple wounds. Forty-one of the 98 wounds were DFUs, 19 were VLUs, 10 were surgical wounds, and 28 were other wounds, including pressure ulcers, arterial wounds, chronic wounds, open hematomas, and gangrenous wounds, among others. The average wound size was 13.3 cm^2^ (median: 3.62 cm^2^) with an average duration of 8.7 months (median: 4.5 months), and 20 wounds (20.4%) had a duration >12 months. Ninety-six point nine percent of wounds were located on the lower extremity and 3.1% were nonlower extremity wounds located on the tailbone, scrotum, and shoulder. Table 1 describes cumulative wound characteristics as well as wound characteristics for each etiology.

**Table 1.** Wound characteristics

	*Cumulative*	*DFU*	*VLU*	*Surgical*	*Other*^a^
Sample size, *n* (%)	98 (100%)	41 (41.8%)	19 (19.4%)	10 (10.2%)	28 (28.6%)
Wound size (cm^2^)					
Mean (SD)	13.3 (29.6)	10.2 (32.6)	8.3 (14.9)	7.9 (10.7)	23.1 (34.9)
Median (range)	3.62 (0.12–209.6)	3.2 (0.12–209.6)	3.0 (1.26–66.5)	3.6 (0.30–32.5)	5.6 (0.15–124.7)
Wound duration (months) (*n* = 93)^b^					
Mean (SD)	8.7 (7.2)	7.2 (6.73)	14.4 (23.0)	4.9 (5.1)	8.5 (8.59)
Median (range)	4.5 (0.75–101)	4.5 (0.75–27)	7.0 (1–101)	3.0 (1–18)	5.5 (1–24)
Wounds >12 months duration, *n* (%)	20 (20.4%)	7 (17.1%)	6 (31.6%)	1 (10.0%)	6 (21.4%)
Wound location, *n* (%)					
Plantar	11 (11.2%)	10 (24.4%)	—	—	1 (3.6%)
Dorsal	9 (9.2%)	5 (12.2%)	—	—	4 (14.3%)
Medial	2 (2.0%)	1 (2.4%)	—	—	1 (3.6%)
Lateral	3 (3.1%)	2 (4.9%)	—	1 (10.0%)	—
Malleolus	19 (19.4%)	1 (2.4%)	11 (57.9%)	2 (20.0%)	5 (17.9%)
Heel	11 (11.2%)	5 (12.2%)	—	—	6 (21.4%)
Toe	21 (21.4%)	13 (31.7%)	—	1 (10.0%)	5 (17.9%)
Leg	14 (14.3%)	—	8 (42.1%)	2 (20.0%)	5 (17.9%)
Other	8 (8.2%)	4 (9.8%)	—	4 (40.0%)	1 (3.6%)

^a^ Other wounds consisted of pressure ulcers, arterial wounds, chronic wounds, open hematomas, gangrenous wounds, radiation necrosis wounds, lymphedema wounds, ischemic wounds, and necrotizing fasciitis.

^b^ Wound durations were missing for five wounds.

DFU, diabetic foot ulcer; SD, standard deviation; VLU, venous leg ulcer.

### Clinical outcomes

**Figure 1.**
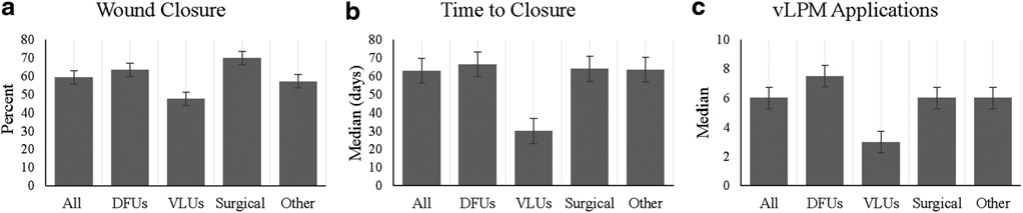
A graphical representation of clinical outcomes with vLPM application plus SOC for **(a)** the proportion of patients who achieved complete wound closure, **(b)** time to closure, and **(c)** number of applications. SOC, standard of care; vLPM, lyopreserved placental membrane containing viable cells.

**Figure 2.**
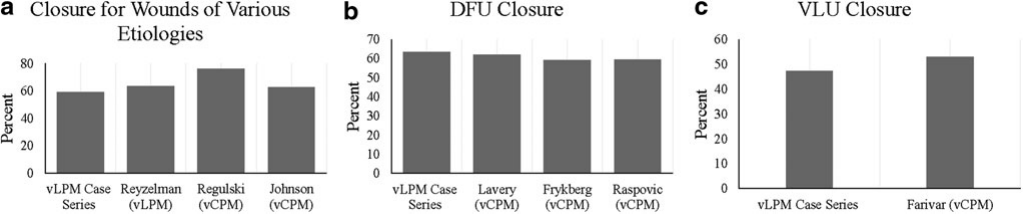
A graphical comparison of closure rates between **(a)** the current study with vLPM, a previous case series utilizing vLPM and previous studies utilizing vCPM in the treatment of wounds of various etiologies, **(b)** the current study and previous studies utilizing vCPM for chronic DFUs, and **(c)** the current study and a previous study utilizing vCPM for chronic VLUs. DFU, diabetic foot ulcer; vCPM, cryopreserved placental membrane containing viable cells; VLU, venous leg ulcer.

Fifty-eight of the 98 wounds (59.2%) that received vLPM applications achieved complete closure with a median time to closure of 63 days and six applications. Approximately 63% of DFUs, 47% of VLUs, 70% of surgical wounds, and 57% of other types of wounds achieved complete wound closure. Clinical outcomes are graphically presented in Fig. 1. Closure rates with vLPM application plus SOC for wounds of different etiologies in the present study are similar to those seen in previous vCPM studies (Fig. 2), with those studies averaging a closure rate of 62.3%.

Forty wounds did not achieve complete closure, of which, only 5 (12.5%) increased in size. The mean PAR for nonclosed wounds was 42.3%. The average size of nonclosed wounds was 19.0 cm^2^ with an average duration of 13.3 months. No AEs were attributable to vLPM application.

Bivariate analyses of the wound closure (primary study endpoint) versus other study variables revealed associations between wound duration and wound closure (*p* = 0.002). Time to closure was associated with a diabetes diagnosis (*p* = 0.03), wound size (*p* = 0.01), and wound duration (*p* = 0.03).

**Figure 3.**
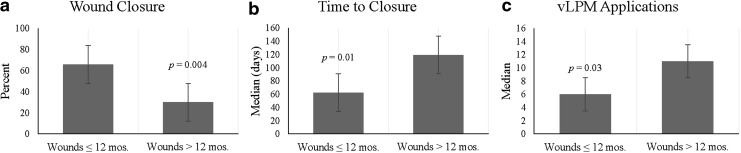
A graphical comparison of clinical outcomes between wounds ≤12 months duration and >12 months duration for **(a)** the proportion of patients who achieved complete wound closure, **(b)** time to closure, and **(c)** number of applications.

A comparison of clinical outcomes between wounds ≤12 months duration and >12 months duration are presented in Fig. 3. There were 73 wounds ≤12 months duration and 20 wounds >12 months duration (missing duration data for 5 wounds). Forty-eight out of 73 wounds (65.8%) that were less than 12 months duration achieved complete closure, while only 6 out of 20 wounds (30.0%) that were greater than 12 months duration achieved complete closure (*p* = 0.004). Significant differences were also seen for time to closure and number of applications between the two subsets of wounds. Wounds ≤12 months duration achieved closure in a median time of 62.5 days with 6.0 applications compared to 119.5 days with 11.0 applications for wounds >12 months duration (*p* = 0.01, *p* = 0.03).

**Figure 4.**
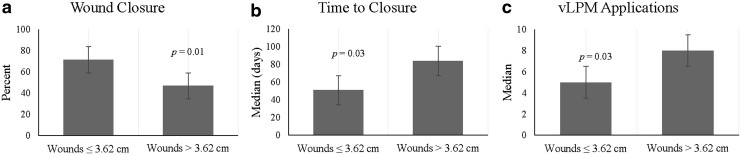
A graphical comparison of clinical outcomes between wounds ≤3.62 and >3.62 cm^2^ for **(a)** the proportion of patients who achieved complete wound closure, **(b)** time to closure, and **(c)** number of applications.

Figure 4 shows the clinical outcome comparisons between wounds ≤3.62 cm^2^ and wounds >3.62 cm^2^. Forty-nine wounds were ≤3.62 cm^2^, of which 71.4% achieved closure compared with 49 wounds >3.62 cm^2^, of which 46.9% achieved complete closure (*p* = 0.01). The smaller wounds, with an average duration of 6.8 months, achieved closure in a median time of 51.0 days and 5.0 applications compared to 84.0 days and 8.0 applications for the larger wounds, which had an average duration of 10.7 months (*p* = 0.03, *p* = 0.03).

## Discussion

The main objective of this multicenter, open-label case series was to evaluate the clinical outcomes of vLPM, a commercially available placental tissue allograft, in the treatment of nonhealing wounds. We further compared wound closure rates achieved with the use of vLPM in this study to those previously reported for vCPM, the cryopreserved formulation of placental membrane. Patients with wounds treated in this study are representative of real-world, hard-to-heal wounds that providers face daily in their practice.

Ninety-eight wounds of various etiologies were treated with vLPM in this study: 41 DFUs, 19 VLUs, 10 surgical wounds, and 28 other wounds. The results of this study demonstrated an overall closure rate of 59.2% in a median time to closure of 63 days and six applications. Clinical outcomes for all wounds as well as outcomes separated by etiology are graphically presented in Fig. 1. Approximately 63% of DFUs, 47% of VLUs, 70% of surgical wounds, and 57% of other types of wounds achieved complete closure.

Clinical outcomes of vCPM have been reported in multiple studies for wounds of various etiologies and locations.^12–20^ In the present study using vLPM adjunct to SOC, we demonstrated similar closure rates to those rates previously shown in clinical studies with the cryopreserved formulation (Fig. 2). Specifically, the 63.4% closure rate for DFUs in this vLPM study is similar to the 62.0%, 59.3%, and 59.4% closure rates previously reported with vCPM in a multicenter randomized controlled trial (RCT) for chronic DFUs, in a prospective multicenter open-label clinical trial for complex chronic DFUs with exposed deep structures, and in a retrospective wound registry study representing real-world patients with DFUs, respectively.^12,14,16^ The closure rate for VLUs of 47.4% is also similar to the 53.0% closure rate in a previous study utilizing vCPM in the treatment of chronic VLUs.^17^

We further performed analyses of associations between study variables and clinical outcomes. Our results of the bivariate analysis revealed that wound closure depends on wound duration before treatment and time to closure is associated with a diabetes diagnosis, wound size, and wound duration. A closer look at the data for wounds ≤12 and >12 months duration showed a significant difference in the proportion of patients who achieved complete wound closure (*p* = 0.004), time to closure (*p* = 0.01), and number of vLPM applications (*p* = 0.03) between the two cohorts (Fig. 3). The majority of wounds in this study are DFUs. The cutoff wound size of 3.62 cm^2^ was selected as it represents both a typical DFU size and aligns with the size of DFUs in other clinical studies.^12–14,16,26^ These data showed significant differences between wounds ≤3.62 cm^2^ and >3.62 cm^2^ for the proportion of patients who achieved complete closure (*p* = 0.01), time to closure (*p* = 0.03), and vLPM applications (*p* = 0.03) (Fig. 4).

Previous DFU and VLU studies have shown evidence of similar prognostic indicators for wound closure. Margolis *et al.* analyzed data from 27,630 DFU patients and evaluated several prognostic models with a wound closure outcome by 20 weeks of care. This study found that wounds greater than 2 cm^2^, greater than 2 months duration, and a wound grade greater than 2 were less likely to close.^26^ In 2015, Fife *et al.* generated a predictive model for wound closure using data from 13,226 patients with DFUs. The results indicated that there are several factors that are associated with wound closure outcomes. Such factors include the presence of infection, first wound area, age, Wagner grades, peripheral vascular disease diagnosis, and wound age at first encounter.^27^

These prognostic indicators are similar to those that have been identified in VLU studies. In a multicenter, double-blind, parallel group study, Phillips *et al.* analyzed 165 patients with a chronic VLU to determine if there were potential prognostic factors for ulcer closure and time to closure. The results showed that ulcers with a smaller baseline area and a shorter duration were more likely to close. For the time to closure endpoint, the results also indicated that baseline ulcer area and duration of the ulcer were both important predictive factors.^28^

Similar results were seen in a larger, retrospective cohort study conducted by Taylor *et al.* In this study, data were analyzed for 325 patients with 345 VLUs. The results showed that closure rates were significantly related to history of previous ulceration, the amount of ulcer exudate, high BMI, a larger total ulcer area, increasing age, and male gender.^29^

The association of wound closure and time to closure with wound size and duration are consistent with what was seen in the current case series. Only 30% of wounds >12 months duration and only 46.9% of wounds >3.62 cm^2^ achieved complete closure. Larger wounds with a longer duration put patients at a high risk for nonclosure as well as wound-related infections, hospitalizations, and amputations, the three major and costly complications associated with open wounds.^30^ These data indicate that larger wounds and wounds open for a longer duration will require more applications of vLPM, take longer to achieve complete closure, and suggest that these sicker, real-world patients could benefit from advanced wound-care modalities such as vCPM or vLPM earlier in their care.

A recently published study that analyzed wound closure rates using U.S. Wound Registry (USWR) and reported in RCTs supports the use of advanced wound-care modalities. Based on the analysis of more than 225,000 wounds in USWR and 48 RCTs with 2,620 control subjects, the study concluded that it is likely that in the real world, among complicated patients, healing rates better than 40.0% are not achievable.^30^ With the use of vLPM, a 59.2% overall closure rate was achieved in this study for difficult-to-heal wounds in patients with multiple comorbidities that negatively affect wound healing.

The limitations of this study are the retrospective study design, the lack of a control group and standardized SOC before vLPM treatment, and the relatively small sample size. Due to the lack of strict exclusion criteria, larger wounds and wounds of longer duration before vLPM treatment were included in this study. This could have potentially contributed to the slightly lower closure rate and slower time to closure in the present study compared to previous studies with vCPM. Strengths of this study include providing outcomes for wounds of different etiologies, sizes, and duration, providing guidance on time to closure, number of vLPM applications required for different wounds, and evidence suggesting clinical equivalency between vLPM and vCPM formulations. To confirm clinical equivalency, however, a larger, randomized, prospective trial that is powered to show significance would be needed.

In summary, this study shows positive clinical outcomes with vLPM use for nonhealing wounds of different etiologies and locations, and shows wound closure rates similar to those previously reported with vCPM. The results support that the clinical performance of vLPM is comparable to that of vCPM.

## Innovation

vCPM has been successfully used in the treatment of nonhealing wounds. However, the requirement of ultralow temperature equipment for storage limits its use to those medical facilities that have such equipment. Using a new lyopreservation technique that allows viable tissues to be stored at room temperature, vLPM has recently been developed. The results of this study show positive clinical outcomes for vLPM in the management of nonhealing wounds of various etiologies and locations that are comparable to those outcomes reported previously for vCPM.

**Key Findings**

- The proportion of patients treated with vLPM who achieved complete wound closure by the end of treatment was 59.2% with a median time to closure of 63 days and 6 applications. There were no vLPM-related AEs.
- Closure rates between the current study with vLPM (59.2%) and previous studies with vCPM (average 62.3%) suggest clinical equivalency between the two formulations.
- Our findings suggest a correlation between wound duration before vLPM treatment and wound closure. Wounds ≤12 months duration and ≤3.62 cm^2^ were more likely to close. In addition, a diabetes diagnosis, wound size, and wound duration all show a correlation with time to closure using vLPM.

## About the Authors

**Charles E. Ananian, DPM,** is practicing in Los Angeles, CA. He graduated with honors from California College Podiatric Medicine in 2002. Having more than 17 years of diverse experiences, Dr. Ananian affiliates with many hospitals, including St. Mary Medical Center, White Memorial Medical Center, Presbyterian Intercommunity Hospital, and Beverly Hospital. **R. Daniel Davis, DPM,** is currently a partner at the Family Foot and Ankle Specialists office in Bridgeport, CT. He is a practicing podiatric physician at the Saint Vincent's Wound Care Centers in Trumbull and Stratford, CT, and is board certified by ABFAS, ABPM, and certified in wound care by CMET. He is a fellow of the APWC and a past president of the APMA. **Eric Johnson, MD,** is currently Medical Director, Wound and Hyperbaric Medicine Department at Bozeman Health, Bozeman, MT. Dr. Johnson is board certified in Family Medicine, has practiced 20 years of emergency medicine, and has been involved in wound and hyperbaric medicine. **Matthew J .Regulski, DPM,** is currently a Partner at Ocean County Foot and Ankle Surgical Associates and has many hospital affiliations. Dr. Regulski has been practicing for over 15 years. **Alexander M. Reyzelman, DPM,** is currently a Professor in the Department of Medicine at the California School of Podiatric Medicine at Samuel Merritt University. He is also the Co-Director of the UCSF Center for Limb Preservation. **Molly C. Saunders, BS,** is currently a Senior Manager of Clinical Operations at Osiris Therapeutics, Inc., She received her BS degree in chemical and biomolecular engineering at Johns Hopkins University, and **Alla Danilkovitch, PhD,** is currently Chief Scientific Officer at Osiris Therapeutics, Inc., Columbia, MD. Dr. Danilkovitch has more than 25 years of a broad biomedical research experience, including stem cells, wound healing, and regenerative medicine product development. Dr. Danilkovitch earned her PhD in cell biology.

## Abbreviations and Acronyms

AATB: American Association of Tissue Banks
AE: adverse event
DFU: diabetic foot ulcer
DM: diabetes mellitus
PAD: peripheral arterial disease
PAR: percent area reduction
SD: standard deviation
SOC: standard of care
USP: United States Pharmacopeia
USWR: U.S. Wound Registry
vCPM: cryopreserved placental membrane containing viable cells
vLPM: lyopreserved placental membrane containing viable cells
VLU: venous leg ulcer

## References

[1] Sen CK, Gordillo GM, Roy S, et al. Human skin wounds: a major and snowballing threat to public health and the economy. Wound Repair Regen 2009;17:763–771 19903300 10.1111/j.1524-475X.2009.00543.x PMC2810192

[2] Järbrink K, Ni G, Sönnergren H, et al. The humanistic and economic burden of chronic wounds: a protocol for a systematic review. Syst Rev 2017;6:15 28118847 10.1186/s13643-016-0400-8 PMC5259833

[3] Frykberg RG, Banks J Challenges in the treatment of chronic wounds. Adv Wound Care (New Rochelle) 2015;4:560–582 26339534 10.1089/wound.2015.0635 PMC4528992

[4] U.S. Department of Health and Human Services; Food and Drug Administration; Center for Drug Evaluation and Research; Center for Biologics Evaluation and Research; Center for Devices and Radiological Health. Guidance for industry: chronic cutaneous ulcer and burn wounds—developing products for treatment. 2006 6 https://www.fda.gov/downloads/drugs/guidances/ucm071324.pdf (last accessed 327, 2019)

[5] Climov M, Bayer LR, Moscoso AV, et al. The role of dermal matrices in treating inflammatory and diabetic wounds. Plast Reconstr Surg 2016;138(3Suppl):148S–157S 27556755 10.1097/PRS.0000000000002652

[6] Hughes OB, Rakoski A, Macquhae F, et al. A review of cellular and acellular matrix products: indications, techniques, and outcomes. Plast Reconstr Surg 2016;138(3 Suppl):138S–147S 27556754 10.1097/PRS.0000000000002643

[7] GrafixPRIME^®^ Package Insert. Columbia, MD: Osiris Therapeutics, Inc., 2019

[8] Duan-Arnold Y, Gyurdieva A, Johnson A, et al. Retention of endogenous viable cells enhances the anti-inflammatory activity of cryopreserved amnion. Adv Wound Care (New Rochelle) 2015;4:523–533 26401419 10.1089/wound.2015.0636 PMC4529089

[9] Duan-Arnold Y, Gyurdieva A, Johnson A, et al. Soluble factors released by endogenous viable cells enhance the antioxidant and chemoattractive activities of cryopreserved amniotic membrane. Adv Wound Care (New Rochelle) 2015;4:329–338 26029483 10.1089/wound.2015.0637 PMC4440986

[10] Duan-Arnold Y, Uveges TE, Gyurdieva A, et al. Angiogenic potential of cryopreserved amniotic membrane is enhanced through retention of all tissue components in their native state. Adv Wound Care (New Rochelle) 2015;4:513–522 26339531 10.1089/wound.2015.0638 PMC4528990

[11] Mao Y, Hoffman T, Singh-Varma A, et al. Antimicrobial peptides secreted from human cryopreserved viable amniotic membrane contribute to its antibacterial activity. Sci Rep 2017;7:13722 29057887 10.1038/s41598-017-13310-6 PMC5651856

[12] Lavery LA, Fulmer J, Shebetka KA, et al. The efficacy and safety of Grafix^®^ for the treatment of chronic diabetic foot ulcers: results of a multi-centre, controlled, randomised, blinded, clinical trial. Int Wound J 2014;11:554–560 25048468 10.1111/iwj.12329 PMC7951030

[13] Regulski M, Jacobstein DA, Petranto RD, et al. A retrospective analysis of a human cellular repair matrix for the treatment of chronic wounds. Ostomy Wound Manage 2013;59:38–43 24334364

[14] Frykberg RG, Gibbons GW, Walters JL, et al. A prospective, multicentre, open-label, single-arm clinical trial for treatment of chronic complex diabetic foot wounds with exposed tendon and/or bone: positive clinical outcomes of viable cryopreserved human placental membrane. Int Wound J 2017;14:569–577 27489115 10.1111/iwj.12649 PMC7950156

[15] Johnson EL, Marshall JT, Michael GM A comparative outcomes analysis evaluating clinical effectiveness in two different human placental membrane products for wound management. Wound Repair Regen 2017;25:145–149 27997744 10.1111/wrr.12503

[16] Raspovic KM, Wukich DK, Naiman DQ, et al. Effectiveness of viable cryopreserved placental membranes for management of diabetic foot ulcers in a real world setting. Wound Repair and Reg 2018;26: 213–220 10.1111/wrr.12635 29683538

[17] Farivar BS, Toursavadkohi S, Monahan TS, et al. Prospective study of cryopreserved placental tissue wound matrix in the management of chronic venous leg ulcers. J Vasc Surg 2019;7:228–233 30621916 10.1016/j.jvsv.2018.09.016

[18] Smedley J, Michael GM, Tamire YG Wound closure in smoking peripheral arterial disease patients with treatment-refractory ulcerations. Int J Low Extrem Wounds 2016;15:360–365 27852883 10.1177/1534734616671639 PMC5207297

[19] Johnson EL, Michael GM, Tamire YG Placental membranes for management of refractory cutaneous sinus tracts of surgical origin: a pilot study. J Am Coll Clin Wound Spec 2016;8:31–38 30276122 10.1016/j.jccw.2017.09.001 PMC6161625

[20] Suzuki K, Michael G, Tamire Y Viable intact cryopreserved human placental membrane for a non-surgical approach to closure in complex wounds. J Wound Care 2016;25:S23–S31 10.12968/jowc.2016.25.Sup10.S25 27681807

[21] Bissoyi A, Kumar A, Rizvanov AA, et al. Recent advances and future direction in lyophilisation and desiccation of mesenchymal stem cells. Stem Cells Int 2016, Article ID 3604203 10.1155/2016/3604203 PMC5002305 27597869

[22] GrafixPL PRIME™ Package Insert. Columbia, MD: Osiris Therapeutics, Inc., 2018

[23] Dhall S, Sathyamoorthy M, Kuang JQ, et al. Properties of viable lyopreserved amnion are equivalent to viable cryopreserved amnion with the convenience of ambient storage. PLoS One 2018;13:e0204060 30278042 10.1371/journal.pone.0204060 PMC6168127

[24] Dhall S, Hoffman T, Sathyamoorthy M, et al. A viable lyopreserved amniotic membrane modulates diabetic wound microenvironment and accelerates wound closure. Adv Wound Care 2019 10.1089/wound.2018.0931 (online ahead of print 3 27, 2019) PMC6657363 31346490

[25] Mao Y, Hoffman T, Dhall S, et al. Endogenous viable cells in lyopreserved amnion retain differentiation potential and anti-fibrotic activity in vitro. Acta Biomater 2019 [Epub ahead of print]; DOI: 10.1016/j.actbio.2019.06.002 31176843

[26] Margolis DJ, Allen-Taylor L, Hoffstad O, Berlin JA Diabetic neuropathic foot ulcers: predicting which ones will not heal. Am J Med 2003;115:627–631 14656615 10.1016/j.amjmed.2003.06.006

[27] Fife CE, Horn SD, Smout RJ, et al. Predictive model for diabetic foot ulcer outcome: the wound healing index. Adv Wound Care (New Rochelle) 2016;5:279–287 27366589 10.1089/wound.2015.0668 PMC4900227

[28] Phillips TJ, Machado F, Trout F, et al. Prognostic indicators in venous ulcers. J Am Acad Dermatol 2000;43:627–630 11004617 10.1067/mjd.2000.107496

[29] Taylor RJ, Taylor AD, Smyth JV Using an artificial neural network to predict healing times and risk factors for venous leg ulcers. J Wound Care 2002;11:101–105 11933726 10.12968/jowc.2002.11.3.26381

[30] Fife CE, Carter MJ, Walker D, Thomson B Wound care outcomes and associated cost among patients treated in US outpatient wound centers: data from the US wound registry. Wounds 2012;24:10–17 25875947

